# Associations between comorbidities and survival outcomes in older adults with cancer: a scoping review of international and Japanese evidence

**DOI:** 10.1186/s12877-026-07891-z

**Published:** 2026-07-22

**Authors:** Yuki Itani, Ayumu Matsuoka, Kyoko Obama, Maiko Fujimori

**Affiliations:** 1 Division of Survivorship Research, National Cancer Center Institute for Cancer Control, Tokyo, Japan; 2 Department of Cancer Survivorship and Digital Medicine, The Jikei University School of Medicine, Tokyo, Japan

**Keywords:** Comorbidity, Cancer survivor, Survival outcome, Older adults, Japan, Scoping review

## Abstract

**Background:**

Advances in cancer treatment have improved survival among older adults with cancer, many of whom experience multiple comorbidities. This scoping review synthesized international and Japanese evidence regarding associations between comorbidities and survival outcomes in older adults with cancer.

**Methods:**

We conducted a two-stage scoping review using PubMed and Ichushi. In the primary analysis, we searched for large-scale population- or registry-based studies globally to examine whether the pathways through which comorbidities influence survival outcomes in older adults with cancer, previously described for diabetes, are similarly applicable to comorbidities in general. In the secondary analysis, we searched for Japan-based studies to examine whether similar associations and mechanisms were observed in smaller clinical cohort settings in Japan. Data extraction focused on comorbidity measures, survival outcomes, and proposed mechanisms linking comorbidities to prognosis, which were categorized according to six conceptual pathways.

**Results:**

Eighteen international studies and 17 Japan-based studies met the eligibility criteria. Across both evidence streams, greater comorbidity burden was consistently associated with poorer overall survival. The Charlson Comorbidity Index was the most frequently used comorbidity measure. International studies more commonly suggested that comorbidities contributed to cancer-related mortality through restricted access to standard treatment (Pathway II). By contrast, Japanese studies more often reported associations with competing non-cancer mortality (Pathway I). Evidence remained limited for mechanisms involving increased malignancy, reduced treatment responsiveness, and delayed cancer detection, highlighting substantial gaps in the literature.

**Conclusions:**

Comorbidities were consistently associated with poorer survival outcomes among older adults with cancer. International and Japanese studies appeared to emphasize different mechanisms linking comorbidities to prognosis; however, these differences should be interpreted cautiously owing to heterogeneity in study design, outcome definitions, and data sources. Further large-scale studies incorporating standardized comorbidity assessment are needed to clarify the mechanisms through which comorbidities affect prognosis and to inform survivorship care for aging cancer populations.

**Supplementary Information:**

The online version contains supplementary material available at https://doi.org/10.1186/s12877-026-07891-z.

## Background

Early detection and treatment have increased the number of older adults who survive for extended periods after a cancer diagnosis [1]. The growing population of cancer survivors reflects both rising cancer incidence and advances in early detection, which increase the number of individuals living with a cancer diagnosis over time, even when cancer-specific mortality rates have not declined proportionally [1]. Older cancer survivors often present with multiple comorbidities that significantly affect their health outcomes [2]. While individual studies have examined associations between specific comorbidities and survival outcomes in older adults with cancer across various countries [3], to our knowledge, no comprehensive review has simultaneously synthesized this evidence across multiple comorbidity types and cancer sites [4]. Existing systematic reviews have focused either on specific comorbidities, such as diabetes [5] or depression [6], or on the association between comorbidity burden and prognosis in specific cancer types, such as colorectal [7] or head and neck cancers [8]. Therefore, a broader evaluation synthesizing evidence across multiple comorbidity types and cancer sites is needed to fully understand their collective impact on cancer prognosis in older adults.

According to previous literature [5], drawing on evidence from diabetes as a representative comorbidity, comorbidities may affect survival outcomes through several pathways: (I) direct mortality from the comorbidity itself, (II) restricted access to standard cancer treatment resulting in cancer-related mortality, (III) increased cancer malignancy, (IV) reduced responsiveness to treatment, (V) delayed cancer detection due to reduced likelihood of timely screening, leading to diagnosis at a more advanced stage, and (VI) other mechanisms. Understanding which pathways are most commonly reported may help clarify how comorbidities influence prognosis in older adults with cancer. Japan has the highest proportion of older adults globally [9] and offers unique insights into comorbidity management. Older adults in Japan frequently experience frailty and multimorbidity [10]. Japan's extraordinary longevity paradoxically coexists with a high prevalence of frailty, which may reduce treatment tolerability and increase competing mortality risk, thereby worsening overall survival in older cancer patients. Furthermore, Japan's healthcare system and clinical practice patterns differ substantially from those of many Western countries, potentially influencing how comorbidities affect treatment access and survival outcomes. However, Japan's National Cancer Registry does not routinely capture comorbidity data, limiting the availability of large-scale registry-based evidence on this topic in Japan. Therefore, synthesizing international registry-based and Japanese clinical cohort evidence is needed to examine the applicability of identified pathways to the Japanese clinical context and to identify Japan-specific patterns relevant to domestic cancer care and global geriatric oncology.

Accordingly, we conducted a scoping review to synthesize evidence on associations between comorbidities and survival outcomes in older adults with cancer. Our objectives were: (1) to map the international evidence on associations between comorbidities and survival outcomes in older adults with cancer, and (2) to examine whether similar associations and mechanisms are observed in Japan.

## Methods

### Review design

We conducted a scoping review in accordance with the Joanna Briggs Institute (JBI) methodological framework [11] and reported findings following the PRISMA-ScR guidelines [12]. A review protocol was developed prior to study selection and is available from the corresponding author upon reasonable request.

This review was designed from the outset with a two-stage search strategy. In the first stage, large-scale population- or registry-based studies were searched globally to examine whether the pathways previously described for diabetes [5] are similarly applicable to comorbidities in general; no Japan-based registry studies were anticipated given the absence of comorbidity data in Japan's National Cancer Registry. In the second stage, Japan-based non-registry clinical studies were searched to examine whether similar associations and mechanisms were observed in smaller clinical cohort settings, including hospital-based cancer registry studies and electronic medical record cohorts.

### Eligibility criteria

Studies were excluded if they: (1) did not involve patients with cancer; (2) did not meet the older adult age criterion; (3) were conference abstracts, correspondence articles, reviews, commentaries, or case reports; or (4) lacked sufficient information regarding comorbidity assessment or survival outcomes.

### Search strategy

**Fig. 1:**
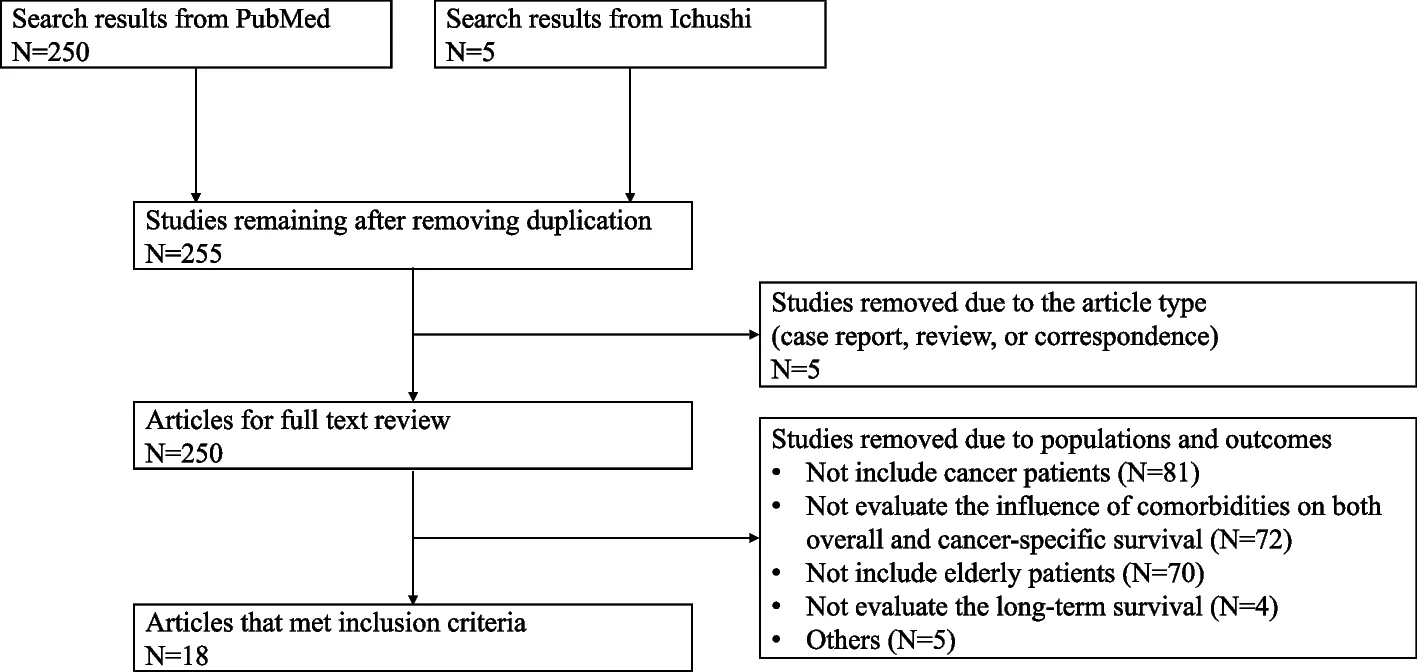
PRISMA flow diagram for study selection (primary analysis): international studies

**Fig. 2:**
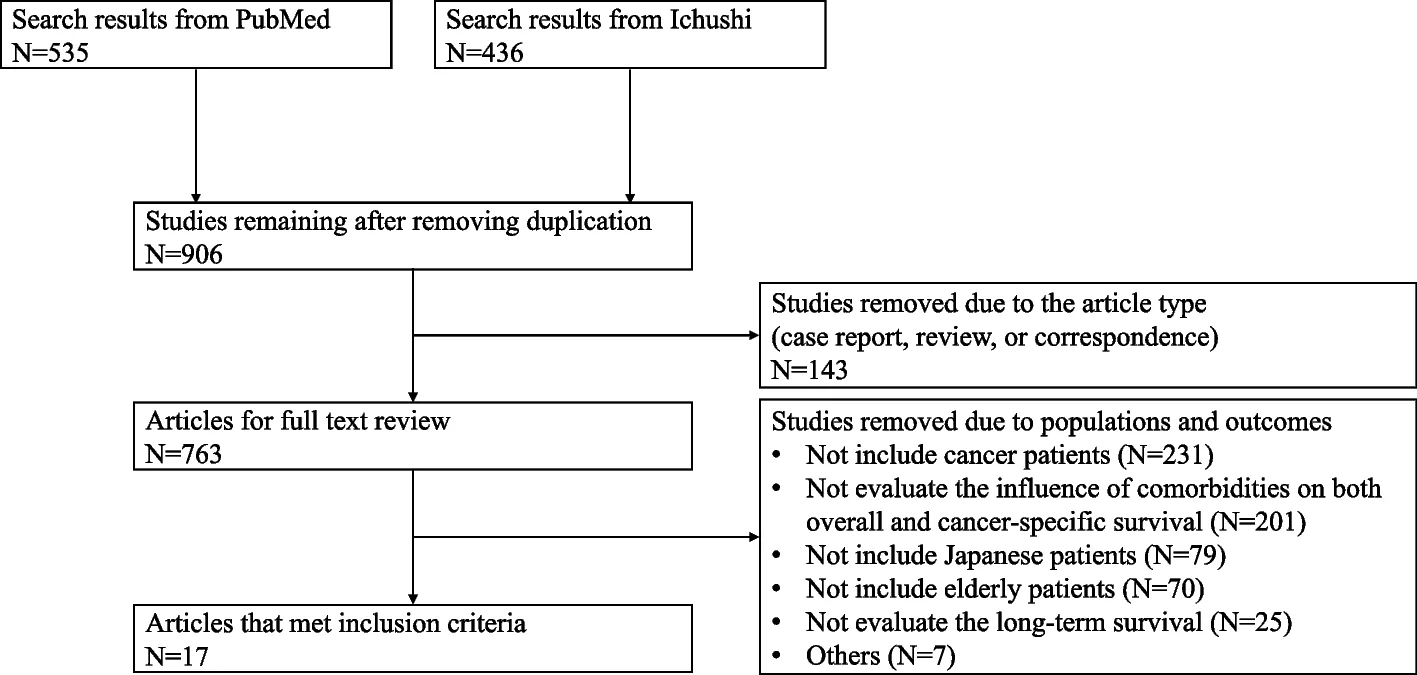
PRISMA flow diagram for study selection (secondary analysis): Japan-based studies

We collaborated with an expert librarian to develop search term combinations and searched for studies published from database inception to November 15, 2024. Searches were conducted in the electronic databases of PubMed and Igaku Chuo Zasshi online (Ichushi), a bibliographic database established in 1903 and maintained by the Japan Medical Abstracts Society, a non-profit, non-governmental organization. Ichushi provides citations and abstracts from approximately 2,500 biomedical journals and serial publications in Japan. We searched PubMed to identify international registry-based studies and Ichushi to identify Japanese-language studies not indexed in PubMed. This dual-database strategy reflected our aim to comprehensively capture both international registry-level evidence and Japan-specific clinical evidence. Three independent reviewers (YI, AM, and KO) independently screened the titles and abstracts of all retrieved records based on predefined inclusion criteria, and achieved an inter-rater agreement of 87.3% for PubMed entries. These reviewers also evaluated the full texts of potentially eligible studies. Any discrepancies during screening or selection were resolved by consensus through discussion. Reasons for full-text exclusions are provided in the PRISMA flow diagram (Figs. 1 and 2).

### Primary analysis

The first-stage search aimed to identify large-scale population- or registry-based studies examining associations between comorbidities and survival outcomes among older adults with cancer, guided by the approach used in a previous review [13]. This stage was designed to examine whether the pathways identified for diabetes as a representative comorbidity [5] are applicable to comorbidities in general, using nationwide registry-level data regardless of country. As anticipated, no Japan-based registry studies were identified, reflecting the absence of standardized comorbidity data in Japan's National Cancer Registry. The search strategy used a combination of MeSH terms and title/abstract keywords, as follows: (“chronic condition” OR “comorbidity” OR “chronic disease”) AND (“cancer” OR “neoplasms”) AND (“mortality” OR “death” OR “survival” OR “survival analysis”) AND (“registry”) AND (“cancer-specific” OR “disease-specific” OR “cause of death”) AND (“Japanese” OR “English”). The complete search strategy is detailed in Tables 1–1 and 1–2 in the Appendix.

We identified 250 articles from PubMed and 5 from Ichushi. After removing duplicates, we excluded case reports, review articles, correspondence, and other non-original publications, leaving 250 articles for full-text review. Ultimately, 18 studies met the inclusion criteria and were included in the primary synthesis (Fig. 1).

No quantitative synthesis or evidence quality evaluation was performed because of the substantial heterogeneity across studies in cancer types, comorbidity definitions, outcome measures, and analytic methods. The results were instead narratively synthesized in accordance with established guidance for scoping reviews.

### Secondary analysis

The second-stage search was designed to examine whether the associations and mechanisms identified in the primary analysis were similarly observed in Japan, extending the evidence to smaller non-registry clinical cohort settings. Registry-related search terms were removed and Japan-specific terms were added, as follows: ("chronic condition" OR "comorbidity" OR "chronic disease") AND ("cancer" OR "neoplasms") AND ("mortality" OR "death" OR "survival" OR "survival analysis") AND ("Japan" OR "Japanese"). The complete search strategy is detailed in Tables 2–1 and 2–2 in the Appendix.

This refined search identified 535 articles from PubMed and 436 from Ichushi. After removing duplicates, we excluded case reports, review articles, correspondence, and other non-original publications. A total of 763 articles were retained for full-text review. Ultimately, 17 studies that examined older adults with cancer in Japan were included in this review (Fig. 2).

### Data extraction and synthesis

Three independent reviewers (YI, AM, and KO) extracted data from the selected studies using predefined criteria. The extracted information included participant characteristics, study focus (concept), setting details (context), methodology, and key findings relevant to the review objectives. Disagreements between reviewers were resolved through discussion, with input from the supervising author (MF) when needed. The extracted data were tabulated using Microsoft Excel.

The survival outcome measures assessed in this review were overall survival (OS), disease-specific survival (DSS), and cancer-specific survival (CSS). OS was defined as the time from the date of diagnosis to either death from any cause or the last follow-up. DSS was defined as the time from the date of diagnosis to death attributable to the disease under study, excluding deaths from unrelated causes. CSS refers to the time from the date of diagnosis to death, specifically attributed to cancer, excluding deaths from non-cancer causes.

Because of substantial heterogeneity across studies in cancer types, comorbidity definitions, outcome measures, and analytical methods, quantitative synthesis was not performed. Findings were instead narratively synthesized, and studies were categorized according to previously proposed conceptual pathways describing how comorbidities may influence survival outcomes [5].

## Results

### Study selection

The first-stage search identified 255 records (250 from PubMed and 5 from Ichushi). After duplicate removal and screening, 18 studies met the eligibility criteria for the international synthesis (Fig. 1).

The second-stage search identified 971 records (535 from PubMed and 436 from Ichushi). After screening and full-text assessment, 17 Japan-based studies were included in the secondary synthesis (Fig. 2).

### Study and patient characteristics

#### International studies

The 18 international studies included a total of 214,217 participants. Sample sizes ranged from 149 in a prostate cancer study [14] to 102,929 in a large-scale lung cancer cohort [15], with data collection spanning 2007 [16] to 2022 [17, 18] (Table 1). Most studies were conducted in North America, including six from the United States [15–17, 19–21] and five from Canada [22–26], with additional studies from Australia, Ireland, Korea, Singapore, Spain, the United Kingdom, and Taiwan. Colorectal cancer was the most frequently studied cancer type [16, 22–24, 27], followed by bladder [21, 25, 26, 28] and prostate [14, 29, 30] cancer. Other studies included multiple cancer types, primarily lung and breast cancer [15, 17–19]. Among the 13 studies that reported cancer stage, several focused on advanced disease, including a large lung cancer cohort in which all participants had Stage IV disease [15].

**Table 1:** Overview of International literature selection on comorbidities and survival outcomes in older adults with cancer

Study	Country	Data source	Design	Participant characteristics	Age characteristics	Cancer type	Cancer stage	Comorbidity grouping	Survival outcome
Du, 2007 [16]	USA	Claims data	Retrospective observational study	18,492 patients aged ≥ 65 with Stage II/III colorectal cancer registered in SEER-Medicare between 1992 and 1999	≥ 65 years (Medicare eligibility); median age: Caucasian 77y, African American 75y, others 76y; proportion aged ≥ 80y: Caucasian 39.8%, African American 30.9%	Colon cancer	II 59.7%, III 40.3%	Claims-based CCI (18 conditions) (0 vs 1 vs 2 vs ≥ 3)	OS, CSS
Howlader, 2014 [19]	USA	Cancer registry data	Retrospective observational study	Patients with lung, breast, prostate, or colon cancer registered in SEER-18 between 2000 and 2009 (~ 28% of US population). Sample size not reported as a single N	No age restriction; analyses performed across age groups: 20–44, 45–54, 55–64, 65–74, 75–84, and ≥ 85 years	Lung, breast, prostate, and colon cancer	NA	Charlson-based score (0 vs > 639 (severe))	OS, CSS
Chen, 2014 [27]	Taiwan	Claims data	Retrospective observational study	6,937 patients with early-stage (Stage I/II) colorectal cancer registered in Taiwan Cancer Registry (2004–2008)	No age restriction; mean age 67.3y (SD 11.9); age distribution: 40–64y 38.7%, ≥ 65y 61.3%; mean age: DM group 70.2y, non-DM group 66.6y	Colon cancer	I 30.9%, II 69.1%	DM, cirrhosis, CHF, CVD, dementia, COPD, RA, hemiplegia, renal disease	OS, CSS
Laccetti, 2015 [15]	USA	Cancer registry data	Retrospective observational study	102,929 patients aged ≥ 65 with Stage IV lung cancer registered in SEER between 1992 and 2009 (14.7% with prior cancer history)	≥ 65 years (SEER-Medicare eligibility); age distribution: < 75y 54.6%, 75–84y 43.2%, ≥ 85y 11.6% (*n* = 102,929)	Lung cancer	IV 100%	CCI (0 vs. ≥ 1)	OS, CSS
Rauh-Hain, 2015 [20]	USA	Claims data	Retrospective observational study	9,042 patients with high-grade endometrial cancer registered in SEER between 1992 and 2009	≥ 66 years (Medicare eligibility); mean age: White 76.2 ± 6.8y, African American 75.1 ± 6.8y	Uterine cancer	NA	CCI (0 vs. 1 vs. ≥ 2)	OS, DSS
Patel, 2015 [28]	Australia	Hospitalization data	Retrospective observational study	5,377 patients with invasive bladder cancer registered in NSW Cancer Registry (2001–2009)	No age restriction; overall 75y; age distribution: < 50y 3.1%, 50–59y 9.1%, 60–69y 20.0%, 70–79y 35.4%, ≥ 80y 32.4%	Bladder cancer	Localized 49.4%, Regional 17.3%, Distant 7.4%, Unknown 25.9%	CCI (0 vs. 1 vs. ≥ 2)	OS, DSS
Williams, 2017 [21]	USA	Claims data	Retrospective observational study	6,582 patients with muscle-invasive bladder cancer (T2–T4a) registered in SEER or Texas Cancer Registry between 2006 and 2011	≥ 66 years (SEER-Medicare); age groups: 66–69, 70–74, 75–79, and ≥ 80 years	Bladder cancer	II 57.0%, III 16.4%, IV 26.4%	CCI (0 vs. 1 vs. 2 vs. ≥ 3)	OS, CSS
Flemming, 2017 [22]	Canada	Claims data	Retrospective observational study	4,326 patients with colorectal cancer who underwent elective surgery in Ontario (2002–2008, Stage I 21%, II 36%, III 38%, IV 5%)	No age restriction; median age 71y (IQR 62–78); 21% of patients received surgery at age ≥ 80y	Colon cancer	I 21%, II 36%, III 38%, IV 5%	CCI (0 vs. ≥ 1)	OS, CSS
Karim, 2017 [23]	Canada	Registry data	Retrospective observational study	6,365 patients with early-stage (Stage I–III) colorectal cancer who underwent elective surgery in Ontario (2002–2008)	No age restriction; mean/median age 70/72y; age distribution: < 60y 18.9%, 60–69y 23.8%, 70–79y 33.4%, ≥ 80y 23.9%	Colon cancer	I 18.3%, II 38.4%, III 43.3%	CCI (0 vs. ≥ 1)	OS, CSS
Merchant, 2017 [24]	Canada	Claims data	Retrospective observational study	2,920 patients with Stage III colorectal cancer who underwent elective surgery in Ontario (2002–2008)	Elderly defined as ≥ 70y (*n* = 1,521, 52%); non-elderly < 70y (*n* = 1,399, 48%); mean age 69y overall	Colon cancer	III 100%	CCI (0 vs. ≥ 1)	OS, CSS
Robinson, 2017 [25]	Canada	Hospitalization data	Retrospective observational study	710 patients with advanced bladder cancer who received palliative chemotherapy in Ontario (1994–2008)	No age restriction; median age 67y; 33% of patients aged ≥ 70y, 10% of patients aged ≥ 80y	Bladder cancer	NA	CCI (0 vs. ≥ 1)	OS, CSS
Siemens, 2017 [26]	Canada	Hospitalization data	Retrospective observational study	2,593 patients with bladder cancer who underwent radical cystectomy in Ontario (2000–2008, 62% received transfusion)	No age restriction; mean age 70.1y; age distribution: 20–59y 15%, 60–69y 24%, 70–79y 41%, ≥ 80y 21%	Bladder cancer	T1 21%, T2 21%, T3 37%, T4 22%	CCI (0 vs. 1 vs. ≥ 2)	OS, CSS
Hah, 2018 [29]	Korea	Cancer registry data	Retrospective observational study	2,253 patients who underwent radical prostatectomy registered in the K-Cap registry (2001–2013)	No age restriction; median age 65y (IQR 60–70y); stratified by age < 70y (*n* = 1,665, 73.9%) vs ≥ 70y (*n* = 588, 26.1%)	Prostate Cancer	II 67.4%, III 32.4%, IV 0.2%	CCI (0 vs. ≥ 1)	OS, CSS
Lee, 2018 [30]	Singapore	Claims data	Retrospective observational study	340 patients with localized high/intermediate-risk prostate cancer treated with observation or pADT in Singapore (2004–2014)	No age restriction; median age 75y (range 46–98y); median age: conservative group 74y (range 46–92y), pADT group 77y (range 49–98y)	Prostate cancer	≤ T2a 70.9%, T2b 2.9%, ≥ T2c 26.2%	CCI (0 vs. 1 vs. 2 vs. ≥ 3)	OS, PCSM
Rezk, 2019 [14]	UK and Northern Ireland	Hospitalization data	Retrospective observational study	149 patients with prostate cancer (Gleason 8–10) who received pADT in London (1999–2006)	No age restriction; mean age 75.5 ± 8.6 years	Prostate cancer	NA	CCI (0 vs. 1 vs. 2 vs. ≥ 3)	OS, CSS
Villavicencio, 2021 [48]	Spain	Hospitalization data	Retrospective observational study	400 CLL patients registered in the Girona Cancer Registry (2008–2016)	No age restriction; median age 72y (range 62–80y); mean age 70.8y (SD 12.2); median age increased with CCI burden: CCI = 0 46y, CCI 1–2 59y, CCI 3–4 74y, CCI > 4 83y	Chronic lymphocytic leukemia	0 56.7%, I–II 15.0%, III–IV 8.7%	CCI (0 vs 1–2 vs 3–4 vs > 4)	OS, CLL-specific mortality
Leach, 2022 [17]	USA	Questionnaire	Retrospective observational study	9,979 long-term cancer survivors who registered in the CPS-II Nutrition Cohort in 1992 and completed the PROMIS questionnaire	≥ 65 years; long-term cancer survivors (5–20 years post-diagnosis); median age 80y	Breast, colorectal, prostate, lung, melanoma, endometrial, non-Hodgkin lymphoma, bladder, kidney, and other cancer	NA	2 or more of DM, heart disease, CVD, Parkinson's disease, COPD, RA	OS, CSS
O'Neill, 2022 [18]	Ireland	Hospitalization data	Retrospective observational study	34,828 cancer patients registered in the Northern Ireland Cancer Registry (2011–2014, 22.6% with CVD history)	No age restriction; age distribution: 0–54y 6,500 (18.7%), 55–69y 10,812 (31.1%), 70–79y 10,541 (30.3%), ≥ 80y 6,975 (20.1%)	Colorectal, lung, female breast, and prostate cancer	I/II 17.3%, III 20.0%, IV 24.2%, Unknown 32.5%	Cardiovascular diseases (10 conditions^1^)	OS, CSS

Congestive heart failure, peripheral vascular disease, arrhythmia (including atrial fibrillation), valvular disease, pulmonary circulation disorders, other ischemic heart disease (including myocardial infarction), venous or arterial embolism and thrombosis, myocarditis, pericarditis, cardiac arrest

*UK* United Kingdom, *USA* United States of America, *NA* Not Available

The Charlson Comorbidity Index (CCI) was the most commonly used measure of comorbidity burden, applied in 13 studies [14, 15, 19–26, 28–30, 48]. Most studies categorized CCI scores dichotomously (CCI = 0 vs. ≥ 1) [15, 22–25, 29, 48], whereas seven studies used multilevel stratifications such as CCI = 0, 1, and ≥ 2 or ≥ 3 [14, 18, 20, 21, 26, 28, 30]. Overall survival (OS) was the most frequently evaluated outcome, followed by cancer-specific survival (CSS) and disease-specific survival (DSS). Survival outcomes were typically assessed using Cox proportional hazards models.

#### Japanese studies

The 17 Japan-based studies covered a wide range of cancer types, including gastric [32–36], colorectal [31, 37–39], bladder [40, 41], breast [39, 42], lung [43, 44], esophageal [45], and laryngeal cancer [46] (Table 2). The largest study examined 4,598 patients with colorectal cancer who underwent curative resection at 22 hospitals across Japan [31]. Several studies focused on early-stage disease, including Stage I lung cancer [43, 44] and breast cancer with 70.9%–80.5% of patients in Stages 0–II [39, 42], whereas others targeted advanced-stage disease such as gastric cancer (53.3% in Stage III) [32] and bladder cancer (35.2%–43.1% in Stages T3–T4) [40, 41].

**Table 2:** Overview of Japanese literature selection on comorbidities and survival outcomes in older adults with cancer

Study	Data source	Design	Participant characteristics	Age characteristics	Cancer type	Cancer stage	Comorbidity grouping	Survival outcome
Inoue, 2015 [43]	Medical record survey	Retrospective observational study	136 patients treated with SBRT at Miyakojima IGRT Clinic (2007–2013)	No age restriction; median age: non-COPD group 77y (range 58–94y), COPD group 76y (range 55–98y)	Lung cancer	I 100%	GOLD stage: 1 (≥ 80%), 2 (50–80%), 3 (30–50%), 4 (< 30%)	OS, CSS
Saito, 2016 [46]	Medical record survey	Retrospective observational study	240 cancer patients treated at University of Tokyo Hospital (2001–2012)	No age restriction; median age 69y (range 37–89y)	Laryngeal cancer	I 35%, II 28%, III 19%, IV 18%	CCI: 0 vs. 1 vs. 2 vs. ≥ 3	OS, CSS
Yamano, 2017 [31]	Medical record survey	Retrospective observational study	4,598 patients who underwent curative resection at 22 hospitals in the JFUP-CRC (2004–2006)	No age restriction; age distribution: < 64y 2,007 (44%), 65–74y 1,614 (35%), ≥ 75y 977 (21%)	Colorectal cancer	I 30%, II 36%, III 34%	CCI continuous	OS, CSS
Shibasaki, 2017 [44]	Medical record survey	Retrospective observational study	268 male cancer patients who underwent thoracoscopic surgery at Jikei University Hospital (2006–2013)	No age restriction; median age: non-COPD group 65y (range 30–84y), COPD group 71y (range 55–84y); proportion aged ≥ 65y: non-COPD 52.3%, COPD 75.6%	Non-small cell lung cancer	IA 64.4%, IB 35.6%	Comorbidity details not reported	OS, CSS
Motoishi, 2018 [37]	Medical record survey	Retrospective observational study	468 patients who underwent complete resection at Kyoto Medical Center (2007–2015)	No age restriction; mean age 70.1y (range 44–88y); elderly subgroup analyses reported separately	Non-small cell lung cancer	IA 41.9%, IB 25.7%, IIA 12.2%, IIB 6.8%, IIIA 13.5%, IIIB 0%	CCI: 0–1 vs. ≥ 2	OS, CSS
Takuwa, 2018 [39]	Medical record survey	Retrospective observational study	286 cancer patients at Shiga Medical Center for Adults (1989–2016)	≥ 70 years (inclusion criterion); compared 70–79y group vs ≥ 80y group	Breast cancer	0 10.0%, I 37.1%, II 33.4%, III 15.2%, IV 3.3%	CCI continuous	OS, DSS
Matsumoto, 2018 [41]	Medical record survey	Retrospective observational study	594 patients who underwent RC at seven Japanese institutions (1990–2013)	No age restriction; median age 67y (IQR 60–73y)	Bladder cancer	T0–T1 35.5%, T2 22.2%, T3 30.3%, T4 12.0%; N0 75.3%, ≥ N1 20.5%	CCI: 0–1 vs. ≥ 2	OS, CSS
Yamashita, 2018 [45]	Other	Retrospective observational study	548 patients who underwent esophagectomy at Cancer Institute Hospital (2009–2014)	No age restriction; median age: low CCI (≤ 1) group 64y (range 32–85y), high CCI (≥ 2) group 68y (range 31–83y)	Esophageal cancer	I 49.3%, II 23.2%, III 24.6%, IV 2.9% (high CCI group)	Presence of comorbidities: hypertension, diabetes, cardiovascular disease, respiratory disease, liver disease, renal disease, brain/mental disease, cancer history, and other	OS, DSS
Nakajo, 2019 [35]	Medical record survey	Retrospective observational study	360 elderly patients treated with ESD at six tertiary hospitals in Japan (2010–2014)	≥ 75 years (inclusion criterion); all 360 patients aged ≥ 75y	Esophageal squamous cell carcinoma	Indication for ESD (early stage)	CCI: 0–2 vs. 3–7; %VC: < 80% vs. ≥ 80%; FEV1.0%: < 70% vs. ≥ 70%	OS, CSS
Iwamoto, 2019 [40]	Medical record survey	Retrospective observational study	253 patients who underwent RARC at 34 institutions in Japan (2009–2017)	No age restriction; mean age: younger < 70y group 62.4y (IQR 39–69y), older ≥ 70y group 76.3y (IQR 70–92y)	Bladder cancer	Tis 4%, T1 or less 15.2%, T2 45.6%, T3 28.8%, T4 6.4%	CCI: 0–1 vs. ≥ 2	OS, CSS
Sugawara, 2020 [36]	Medical record survey	Retrospective observational study	402 patients who underwent curative resection at the University of Tokyo Hospital (2002–2016)	≥ 75 years (inclusion criterion); all 402 patients aged ≥ 75y	Gastric cancer	I 59.5–64.7%, II 16.7–25.5%, III 15.6–19.1%	Presence of comorbidities: hypertension, dyslipidemia, diabetes, obesity, renal dysfunction, anemia, dementia, osteoporosis, sarcopenia, and stroke	OS, CSS
Sakamoto, 2020 [38]	Medical record survey	Retrospective observational study	526 patients who underwent radical resection at Kumamoto University (2005–2016)	No age restriction; young group < 75y (n = 361) vs elderly group ≥ 75y (n = 165)	Colorectal cancer	I 34%, II 42%, III 24% (older patients' group)	CCI: ≤ 3 vs. ≥ 4	OS, CSS
Ishii, 2020 [47]	Medical record survey	Retrospective observational study	961 cancer patients diagnosed between 2009 and 2014	No age restriction; median age 69y (IQR 59–79y); proportion aged ≥ 75y 37.9%; CKD group significantly older: median 80y (IQR 73–85y) vs non-CKD 67y (IQR 58–77y)	Gastrointestinal, hepatobiliary and pancreatic, lung, breast, kidney and urinary tract, and gynecological cancers	IV 100%	CCI: ≤ 2 vs. ≥ 3	OS, CSS
Ishido, 2021 [33]	Medical record survey	Retrospective observational study	358 cancer patients treated with ESD at Kitasato University (2005–2018)	No age restriction; median age: elderly ≥ 75y group 79y (range 76–91y), young ≤ 74y group 69y (range 39–75y)	Esophageal squamous cell carcinoma	Indication for ESD (early stage)	CKD stage: G1–2 (≥ 60), G3a (45–60), G3b (30–45), G4–5 (< 30)	OS, CSS
Mikami, 2021 [34]	Medical record survey	Retrospective observational study	226 patients who underwent LG at Kobe Medical Center West Hospital (2010–2016)	No age restriction; median age: non-elderly ≤ 79y group 69y (range 33–79y), very elderly ≥ 80y group 83y (range 80–89y)	Gastric cancer	I 67.6%, II 17.0%, III 15.4%	CKD only (eGFR < 60)	OS, CSS
Fujimoto, 2022 [32]	Medical record survey	Retrospective observational study	122 patients who underwent curative laparoscopic gastrectomy for advanced gastric cancer at Teikyo University Hospital (2013–2018)	No age restriction; mean age: very elderly ≥ 80y group 84.1 ± 3.4y, control < 80y group 63.5 ± 12.0y	Gastric cancer	II 46.7%, III 53.3%	ACE-27 classification	OS, CSS
Iida, 2022 [42]	Medical record survey	Retrospective observational study	190 cancer patients treated at Takaoka Municipal Hospital (2009–2019)	No age restriction; median age: > 80y group 83.5y (range 80–98y), control group (55–64y) 60.0y (range 55–64y)	Breast cancer	0 3.2%, I 40.3%, II 27.4%, III 27.4%, IV 1.6%	GOLD classification (COPD only vs. COPD +)	OS, CSS

*NA* Not Available

Nine studies used the CCI to assess comorbidity burden [31–37, 40, 45], with patients commonly stratified into low-score (CCI 0–1) and high-score (CCI ≥ 2) groups [33, 35, 45]. Respiratory comorbidities were a particular concern in lung cancer studies [43, 44], and chronic kidney disease was frequently reported in studies of advanced-stage cancers [41, 47].

### Impact of comorbidities on survival outcomes

#### International studies

Most international studies reported that greater comorbidity burden was associated with poorer overall survival among older adults with cancer, with several studies additionally demonstrating associations with worse cancer-specific survival. Karim et al. [23] analyzed 6,365 patients with colon cancer (Stages I–III) and identified significant differences in both OS and CSS. Laccetti et al. [15] investigated 102,929 Stage IV lung cancer patients, and Hah et al. [29] studied 2,253 prostate cancer patients (Stages II–IV), both demonstrating similar associations between comorbidity burden and survival outcomes. Studies using multilevel CCI categorizations showed consistent findings: Siemens et al. [26] and Patel et al. [28] found significant differences in OS and CSS across CCI categories in bladder cancer patients, and Lee et al. [30] and Williams et al. [21] demonstrated similar patterns in prostate and bladder cancer, respectively. O'Neill et al. [18], using a broad cardiovascular comorbidity definition, identified significant differences in both OS and CSS across 34,828 patients with colorectal, lung, breast, or prostate cancer.

#### Japanese studies

Most Japanese studies similarly reported associations between greater comorbidity burden and poorer overall survival. Notably, several studies found that comorbidities were associated with reduced OS without corresponding differences in cancer-specific survival, suggesting that competing non-cancer mortality substantially contributes to prognosis in older Japanese adults with cancer.

Yamano et al. [31] examined 4,598 patients with Stage I–III colorectal cancer and identified significant OS differences across CCI categories. Among studies of esophageal cancer, Ishido et al. [33], Nakajo et al. [35], and Yamashita et al. [45] all reported significant OS differences associated with higher CCI scores, without corresponding effects on CSS. Iwamoto et al. [40] and Motoishi et al. [37] similarly found significant OS differences without CSS effects in bladder and lung cancer patients, respectively. Studies using non-CCI comorbidity measures, including Sugawara et al. [36], who used pulmonary function parameters, and Shibasaki et al. [44], who applied the GOLD classification for COPD, reported OS differences without CSS effects, further supporting the role of competing non-cancer mortality. Sakamoto et al. [38] reported OS differences in older colorectal cancer patients without significant CSS effects, whereas Iida et al. [42] and Takuwa et al. [39] reported comparable cancer-specific survival across age groups despite significant OS differences in breast cancer patients aged ≥ 80 years.

### Pathway classification

#### International studies

Among the international studies, pathway (II), in which comorbidities restrict access to standard cancer treatment and contribute to cancer-related mortality, was most frequently identified (*n* = 8) [15, 18, 21, 23, 26, 28–30] (Table 3). Five studies were classified under pathway (I), representing direct mortality from comorbidities themselves [16, 19, 20, 24, 27]. One study was classified under pathway (V), involving delayed cancer detection [22], and one under pathway (VI), representing broader age-related health deficits including functional status and mental health [17]. No studies primarily examined pathways (III) or (IV). Overall, pathway (II) was most frequently represented, followed by pathway (I), indicating that restricted access to standard treatment and competing mortality are the most commonly investigated mechanisms in international studies.

**Table 3:** Pathways linking comorbidities to survival outcomes in international studies

Study	Comorbidity grouping	Main survival outcome (OS, ACM)	Cancer-specific survival outcome (BCSS, CSS, DSS)	Pathway†					
				**I**	**II**	**III**	**IV**	**V**	**VI**
Du, 2007 [16]	Claims-based CCI (18 conditions) (0 vs 1 vs 2 vs ≥ 3)	Significant difference (all-cause mortality)	No significant difference (cancer-specific mortality)	x					
Chen, 2014 [27]	Charlson-based score (0 vs > 639 (severe))	Significant difference	No significant difference (CSS)	x					
Howlader, 2014 [19]	DM, cirrhosis, CHF, CVD, dementia, COPD, RA, hemiplegia, renal disease	Significant difference (OS)	Significant difference (cancer death, localized/regional)	x					
Rauh-Hain, 2015 [20]	CCI (0 vs. ≥ 1)	Significant difference (OS)	No significant difference (DSS)	x					
Merchant, 2017 [24]	CCI (0 vs. 1 vs. ≥ 2)	Significant difference (OS)	No significant difference (CSS)	x					
Laccetti, 2015 [15]	CCI (0 vs. 1 vs. ≥ 2)	Significant difference (OS)	Significant difference (CSS)		x				
Patel, 2015 [28]	CCI (0 vs. 1 vs. 2 vs. ≥ 3)	Significant difference (OS)	Significant difference (DSS)		x				
Karim, 2017 [23]	CCI (0 vs. ≥ 1)	Significant difference (OS)	Significant difference (CSS)		x				
Siemens, 2017 [26]	CCI (0 vs. ≥ 1)	Significant difference (OS)	Significant difference (CSS)		x				
Williams, 2017 [21]	CCI (0 vs. ≥ 1)	Significant difference (OS)	Significant difference (CSS)		x				
Hah, 2018 [29]	CCI (0 vs. ≥ 1)	Significant difference (all-cause mortality)	Significant difference (CSS)		x				
Lee, 2018 [30]	CCI (0 vs. 1 vs. ≥ 2)	Significant difference (all-cause mortality)	Significant difference (PCSM)		x				
O'Neill, 2022 [18]	CCI (0 vs. ≥ 1)	Significant difference (all-cause mortality) (adjusted for cardiac disease and other comorbidities)	Significant difference (cancer-specific mortality)		x				
Flemming, 2017 [22]	CCI (0 vs. 1 vs. 2 vs. ≥ 3)	Significant difference (all-cause mortality) CCI = 0 vs. CCI ≥ 1 HR1.63 (1.46–1.81)	Significant difference (cancer-specific mortality) CCI = 0 vs. CCI ≥ 1 HR1.35 (1.17–1.56)					x	
Leach, 2022 [17]	CCI (0 vs. 1 vs. 2 vs. ≥ 3)	Significant difference (all-cause mortality)	Significant difference (cancer-specific mortality)						x
Robinson, 2017 [25]	CCI (0 vs 1–2 vs 3–4 vs > 4)	No significant difference (OS)	No significant difference (CSS)	NA	NA	NA	NA	NA	NA
Rezk, 2019 [14]	2 or more of DM, heart disease, CVD, Parkinson's disease, COPD, RA	No significant difference (OS)	No significant difference (CSS)	NA	NA	NA	NA	NA	NA
Villavicenci, 2021	Cardiovascular diseases (10 conditions^1^)	Significant difference (CCI continuous)	No significant difference (CLL-related mortality)	NA	NA	NA	NA	NA	NA

Congestive heart failure, peripheral vascular disease, arrhythmia (including atrial fibrillation), valvular disease, pulmonary circulation disorders, other ischemic heart disease (including myocardial infarction), venous or arterial embolism and thrombosis, myocarditis, pericarditis, cardiac arrest

*BCSS* breast cancer specific survival, *CSS* cancer-specific survival, *DFS* disease-free survival, *DSS* disease-specific survival, *LFS* leukemia-free survival, *LRFS* locoregional recurrence free survival, *NRM* non-relapse mortality, *OS* overall survival, *PFS* progression-free survival, *RFS* recurrence free survival, *RR* recurrence rate, *SSCM* site-specific cancer mortality, *NA* Not Available

†Pathways of comorbidities on survival outcomes: I. die from comorbidity, II. die from cancer without standard treatment, III. die from cancer due to a worse prognosis, IV. die from cancer due to poor response, V. die from cancer due to later diagnosis, VI. Others

#### Japanese studies

In contrast, the majority of Japanese studies were classified under pathway (I) (*n* = 11) [31, 33, 35–40, 42, 44, 45], suggesting that comorbidities frequently contributed to non-cancer mortality without clear increases in cancer-specific mortality (Table 4). Two studies were classified under pathway (II) [32, 34], two under pathway (III) [41, 47], and one under pathway (IV) [46]. No studies were classified under pathways (V) or (VI).

**Table 4:** Pathways linking comorbidities to survival outcomes in Japanese studies

Study	Comorbidity grouping	Main survival outcome (OS, ACM)	Cancer-specific survival outcome (BCSS, CSS, DSS)	Pathway†					
				**I**	**II**	**III**	**IV**	**V**	**VI**
Shibasaki, 2017 [44]	GOLD stage: 1 (≥ 80%), 2 (50–80%), 3 (30–50%), 4 (< 30%)	Significant difference: Disadvantage in COPD group	No significant difference (with or without COPD)	x					
Yamano, 2017 [31]	CCI: 0 vs. 1 vs. 2 vs. ≥ 3	Significant difference: Disadvantage in patients with comorbidity ≥ 1	No significant difference (comorbidity ≥ 1)	x					
Motoishi, 2018 [37]	CCI continuous	Significant difference: Disadvantage in high HbA1c; No significant difference in DM	No significant difference	x					
Takuwa, 2018 [39]	Comorbidity details not reported	Significant difference: Disadvantage in patients aged ≥ 80	No significant difference (age ≥ 80)	x					
Yamashita, 2018 [45]	CCI: 0–1 vs. ≥ 2	Significant difference: Disadvantage in patients with high CCI	No significant difference	x					
Iwamoto, 2019 [40]	CCI continuous	Significant difference: Disadvantage in patients aged ≥ 70	No significant difference (age ≥ 70)	x					
Nakajo, 2019 [35]	CCI: 0–1 vs. ≥ 2	Significant difference: Disadvantage in high lymph node risk group over CCI 2	No significant difference	x					
Sakamoto, 2020 [38]	Presence of comorbidities: hypertension, diabetes, cardiovascular disease, respiratory disease, liver disease, renal disease, brain/mental disease, cancer history, and other	Significant difference: Disadvantage in patients aged ≥ 75 vs. < 75	No significant difference	x					
Sugawara, 2020 [36]	CCI: 0–2 vs. 3–7; %VC: < 80% vs. ≥ 80%; FEV1.0%: < 70% vs. ≥ 70%	Significant difference: Disadvantage in patients with low %VC	No significant difference	x					
Ishido, 2021 [33]	CCI: 0–1 vs. ≥ 2	Significant difference: Disadvantage in patients aged ≥ 75; CCI ≥ 2 affects OS in this age group	No significant difference (age ≥ 75)	x					
Iida, 2022 [42]	Presence of comorbidities: hypertension, dyslipidemia, diabetes, obesity, renal dysfunction, anemia, dementia, osteoporosis, sarcopenia, and stroke	Significant difference: Disadvantage in patients aged > 80	No significant difference (age ≥ 80)	x					
Mikami, 2021 [34]	CCI: ≤ 3 vs. ≥ 4	Significant difference: Disadvantage in patients aged > 80	Significant difference: Disadvantage in patients aged > 80		x				
Fujimoto, 2022 [32]	CCI: ≤ 2 vs. ≥ 3	Significant difference: Disadvantage in patients with CCI ≥ 3	Significant difference: Disadvantage in patients aged > 80 with CCI ≥ 3		x				
Matsumoto, 2018 [41]	CKD stage: G1–2 (≥ 60), G3a (45–60), G3b (30–45), G4–5 (< 30)	Significant difference: Disadvantage increases with CKD severity and age	Significant difference: CKD; No significant difference for age			x			
Ishii, 2020 [47]	CKD only (eGFR < 60)	Significant difference: Disadvantage with CKD (vs. non-CKD); CKD is an independent risk factor	Significant difference: CKD remains associated with CSS/SSCM after multivariable adjustment			x			
Saito, 2016 [46]	ACE-27 classification	Significant difference: High ACE-27 is a poor prognostic factor	Significant difference: High ACE-27 is a poor prognostic factor				x		
Inoue, 2015 [43]	GOLD classification (COPD only vs. COPD +)	No significant difference (with or without COPD)	No significant difference (with or without COPD)	NA	NA	NA	NA	NA	NA

*BCSS* breast cancer specific survival, *CSS* cancer-specific survival, *DFS* disease-free survival, *DSS* disease-specific survival, *LFS* leukemia-free survival, *LRFS* locoregional recurrence free survival, *NRM* non-relapse mortality, *OS* overall survival, *PFS* progression-free survival, *RFS* recurrence free survival, *RR* recurrence rate, *SSCM* site-specific cancer mortality, *NA* Not Available

^†^Pathways of comorbidities on survival outcomes: I. die from comorbidity, II. die from cancer without standard treatment, III. die from cancer due to a worse prognosis, IV. die from cancer due to poor response, V. die from cancer due to later diagnosis, VI. Others

These findings suggest that comorbidities are consistently associated with poorer survival outcomes in older adults with cancer across both evidence streams. Notably, pathways (III) and (IV), representing increased cancer malignancy and reduced treatment responsiveness, were not identified in any international registry-based study, suggesting that these mechanisms remain understudied in large-scale registry-based research, potentially owing to limitations in the granularity of registry data. Similarly, pathways (V) and (VI) were absent from all Japan-based studies, indicating that delayed cancer detection and broader age-related health deficits have not been examined in Japan-based clinical cohort research. These gaps highlight important areas for future investigation in both settings.

## Discussion

This scoping review synthesized international and Japanese evidence regarding associations between comorbidities and survival outcomes in older adults with cancer. Across both international and Japan-based studies, greater comorbidity burden was consistently associated with poorer overall survival. These findings support previous reports suggesting that comorbidities substantially influence prognosis in older adults with cancer [5, 7, 8]. Although the CCI was frequently used, its application varied across studies. Accordingly, this review does not aim to estimate the relative magnitude of risk associated with individual comorbidities, but rather to summarize the directions and conceptual mechanisms of their associations with survival outcomes.

A notable finding of this review was not only which pathways were commonly reported, but also which pathways were entirely absent from each evidence stream. Pathways (III) and (IV) were not identified in any international registry-based study, while pathways (V) and (VI) were absent from all Japan-based studies, suggesting that the scope of comorbidity research differs substantially between these two settings. International registry-based studies more frequently identified pathway (II), in which comorbidities restrict access to standard cancer treatment, while Japan-based studies more often identified pathway (I), reflecting competing non-cancer mortality. However, these observations should be interpreted in light of the substantial differences in study design and data sources between the two evidence streams. Several Japanese studies have underscored the importance of assessing comorbidities in this context [52]. This pattern is consistent with findings from a Japanese population-based study examining competing causes of death, which demonstrated that older adults with low-lethality cancers tended to die from non-cancer causes, whereas those with advanced lung cancer predominantly died of cancer within five years [53]. These findings are consistent with previous reviews focusing on specific cancer types, which demonstrated associations between comorbidity burden and OS in colorectal and pharyngeal cancers [7, 49, 50], while showing no associations with disease-free survival.

However, these differences should be interpreted cautiously. The international literature largely consisted of large-scale population- or registry-based studies that could distinguish cancer-specific and non-cancer mortality, whereas the Japanese literature primarily consisted of hospital-based cohort studies with smaller sample sizes and heterogeneous outcome definitions. Therefore, the observed differences may reflect variations in study design, patient selection, and data availability rather than true country-level differences in the mechanisms linking comorbidities and survival outcomes.

Systemic and cultural factors may also contribute to the observed patterns. In many countries outside Japan, systemic barriers such as insurance limitations and financial constraints often restrict access to cancer treatment [54]. However, clinical guidelines in these settings often emphasize patient frailty rather than chronological age, allowing fit patients with comorbidities to receive standard care [55]. In contrast, although Japan's universal health coverage facilitates access to care, national clinical guidelines tend to adopt a conservative stance, often excluding older adults with comorbidities from standard treatment. Their frequent exclusion from clinical trials may further limit the evidence base, reinforcing conservative recommendations. These factors in treatment access, eligibility, and clinical decision-making likely contribute to the pathway patterns observed in this review, reflecting not only biological vulnerabilities but also broader variations in medical practice.

The findings highlight the importance of comorbidity assessment in geriatric oncology. Japan represents a particularly important setting for studying these issues because of its rapidly aging population and increasing prevalence of multimorbidity. However, large-scale registry-based studies incorporating comorbidity data remain limited in Japan, primarily because national cancer registry data do not routinely include comorbidity information [51]. One potential approach to address this gap would be to link cancer registry data with existing administrative databases. For example, the Diagnosis Procedure Combination (DPC) system [56]—a patient classification system used for hospitalized patients in Japan—contains detailed clinical information that could complement cancer registry data and enable more comprehensive evaluations of comorbidities and survival outcomes. Advancing survivorship care for older adults will ultimately require interdisciplinary collaboration to effectively address comorbidities.

This scoping review has several limitations. First, the lack of large-scale registry-based studies in Japan weakens the overall strength of the evidence, and the potential for publication bias may have influenced the findings. Second, the literature search was restricted to PubMed and Ichushi and did not include major databases such as Embase or Cochrane Library, potentially resulting in the omission of relevant studies. Additionally, relevant Japanese registry-based studies may have been missed if comorbidity information was not captured as a MeSH term. Future systematic reviews should incorporate broader database coverage to minimize this limitation. Third, the definition and measurement of comorbidities varied considerably across studies. While some used the CCI, others focused on specific conditions, and among those who did, scoring approaches differed substantially. Furthermore, age reporting varied considerably across included studies. Several studies did not explicitly report the mean or median age of participants or the proportion aged ≥ 65 years, which may limit the confidence with which findings can be attributed specifically to older adults. Although all included studies met our predefined age eligibility criteria, the incomplete reporting of age-related characteristics in some primary studies may affect the interpretation and generalizability of the findings to the broader population of older adults with cancer. Fourth, heterogeneity in cancer type and disease severity limited the comparability and generalizability of findings across studies. Finally, because this was a scoping review, formal quality appraisal and meta-analysis were not performed [57]. Accordingly, the findings should be interpreted as descriptive and hypothesis-generating rather than causal, and do not permit inferences regarding whether improved comorbidity management can modify survival outcomes.

Despite these limitations, this review provides a structured overview of how comorbidities may influence survival outcomes in older adults with cancer and highlights important gaps in the current evidence base, particularly in Japan. Further large-scale studies incorporating standardized comorbidity assessment are needed to clarify the mechanisms through which comorbidities affect prognosis and to inform survivorship care for aging cancer populations globally.

## Conclusion

Comorbidities were consistently associated with poorer survival outcomes among older adults with cancer across both international and Japanese studies. International evidence more frequently suggested that comorbidities restrict access to standard cancer treatment and contribute to cancer-related mortality, whereas Japanese studies more often indicated that older adults with cancer die directly from comorbidities rather than from cancer itself. However, these patterns should be interpreted cautiously because study designs, outcome definitions, and data sources differed substantially between international and Japan-based evidence. Evidence remains limited for mechanisms involving increased malignancy, reduced treatment responsiveness, and delayed cancer detection, highlighting substantial gaps in the literature. To our knowledge, this review represents the first structured synthesis of international registry-based and Japanese clinical evidence on the impact of comorbidities on survival outcomes in older adults with cancer, highlighting both shared patterns and important differences that warrant further investigation. Further large-scale studies incorporating standardized comorbidity assessment are needed to better clarify the mechanisms linking comorbidities and survival outcomes in aging cancer populations.

## Supplementary Information

Supplementary Material 1.

## Data Availability

All data generated or analyzed during this study are included in this published article and its supplementary information files. The study protocol and data extraction forms are available from the corresponding author upon reasonable request.

## Declarations

This review was not registered. The protocol is available from the corresponding author upon request.

## Glossary

Charlson Comorbidity Index (CCI): A widely used scoring system that predicts mortality by classifying and weighting comorbid conditions.
Overall Survival (OS): The time from diagnosis to death from any cause or to the last follow-up.
Disease-Specific Survival (DSS): The time from diagnosis to death attributable to the disease under study, excluding deaths from unrelated causes.
Cancer-Specific Survival (CSS): The time from diagnosis to death caused specifically by cancer, excluding deaths from non-cancer causes.
Diagnosis Procedure Combination (DPC): A patient classification system used in Japan to categorize acute-phase hospitalizations for quality improvement and health services research.
Frailty: A geriatric syndrome characterized by decreased physiological reserves and increased vulnerability to adverse health outcomes.
Propensity Score Matching (PSM): A statistical method that attempts to reduce bias by matching participants with similar baseline characteristics across treatment groups.
Joanna Briggs Institute (JBI) Framework: A methodological guideline for conducting systematic and scoping reviews.
PRISMA-ScR: Preferred Reporting Items for Systematic Reviews and Meta-Analyses Extension for Scoping Reviews; a standardized reporting guideline for scoping reviews.
