# Supplementary material for: Associations between comorbidities and survival outcomes in older adults with cancer: a scoping review of international and Japanese evidence
Source: BMC Geriatr. 2026 Jul 22;26:1309. doi: 10.1186/s12877-026-07891-z (PMC13681871; doi:10.1186/s12877-026-07891-z)
Supplement: Supplementary file 1 [file 12877_2026_7891_MOESM1_ESM.docx]

**Table 1-1 PubMed Search Queries: Registry**

| No | Query |
| --- | --- |
| #1 | ("Chronic conditions" [TIAB] OR "Chronic condition" [TIAB] OR comorbidit* [TIAB] OR "Chronic disease [MH]"） |
| #2 | AND (cancer [TIAB] OR neoplas* [TIAB] OR Neoplasms [MH]) |
| #3 | AND (Mortality [MH] OR death [MH] OR survival [MH] OR "survival analysis" [MH]) |
| #4 | AND registr*[TIAB] |
| #5 | AND ("cancer-specific" [TIAB] OR "disease-specific" [TIAB] OR "cause* of death"[TIAB]) |
| #6 | AND (JAPANESE[LA] OR ENGLISH[LA]) |

**Table 1-2 Ichushi Search Queries: Registry**

| No | Terms in Japanese | Terms translated in English |
| --- | --- | --- |
| #1 | [mansei shikkan]/TH | [Chronic disease]/TH |
| #2 | mansei shikkan/TA | Chronic disease/TA |
| #3 | #1 or #2 | #1 or #2 |
| #4 | shuyou/TH | Tumor/TH |
| #5 | shuyou/TA | Tumor/TA |
| #6 | gan/TA | Cancer/TA |
| #7 | #4 or #5 or #6 | #4 or #5 or #6 |
| #8 | "shibou"/TH | "Death"/TH |
| #9 | "shibouritsu"/TH | "Mortality rate"/TH |
| #10 | [seizon]/TH | [Survival]/TH |
| #11 | [seizonritsu]/TH | [Survival rate]/TH |
| #12 | #8 or #9 or #10 or #11 | #8 or #9 or #10 or #11 |
| #13 | #3 and #7 and #12 | #3 and #7 and #12 |
| #14 | [touroku]/TH | [Registration]/TH |
| #15 | gan touroku/TA | Cancer registration/TA |
| #16 | #14 or #15 | #14 or #15 |
| #17 | gan tokui/TA | Cancer-specific/TA |
| #18 | shikkan tokui/TA | Disease-specific/TA |
| #19 | "shiin"/TH | "Cause of death"/TH |
| #20 | shiin/TA | Cause of death/TA |
| #21 | #17 or #18 or #19 or #20 | #17 or #18 or #19 or #20 |
| #22 | #13 and #16 and #21 | #13 and #16 and #21 |

**Table 2-1** **PubMed Search Queries: Japanese Non-Registry**

| No | Query |
| --- | --- |
| #1 | ("Chronic conditions" [TIAB] OR "Chronic condition" [TIAB] OR comorbidit* [TIAB] OR "Chronic disease [MH]"） |
| #2 | AND (cancer [TIAB] OR neoplas* [TIAB] OR Neoplasms[MH]) |
| #3 | AND (Mortality [MH] OR death [MH] OR survival [MH] OR "survival analysis" [MH]) |
| #4 | AND (JAPAN[PL] OR Japanese [LA] OR JAPAN*[Tiab]) |

**Table 2-2 Ichushi Search Queries: Japanese Non-Registry**

| No | Terms in Japanese | Terms translated in English |
| --- | --- | --- |
| #1 | (kyouzon shikkan/TH or heizon-shou/AL) | (Coexisting disease/TH or Comorbidity/AL) |
| #2 | (mansei shikkan/TH or mansei shikkan/AL) | (Chronic disease/TH or Chronic disease/AL) |
| #3 | #1 or #2 | #1 or #2 |
| #4 | shuyou/TH | Tumor/TH |
| #5 | gan/AL | Cancer/AL |
| #6 | shuyou/AL | Tumor/AL |
| #7 | shuyou/TA | Tumor/TA |
| #8 | gan/TA | Cancer/TA |
| #9 | #4 or #5 or #6 or #7 or #8 | #4 or #5 or #6 or #7 or #8 |
| #10 | shibou/TH | Death/TH |
| #11 | seizon/TH | Survival/TH |
| #12 | (seizonsha/TH or gan sabaibaa/TH) | (Survivor/TH or Cancer survivor/TH) |
| #13 | shibouritsu/TH | Mortality rate/TH |
| #14 | seizon bunseki/TH | Survival analysis/TH |
| #15 | seizon bunseki/TA | Survival analysis/TA |
| #16 | shibouritsu/TA | Mortality rate/TA |
| #17 | #10 or #13 or #16 | #10 or #13 or #16 |
| #18 | #11 or #12 or #14 or #15 | #11 or #12 or #14 or #15 |
| #19 | #17 or #18 | #17 or #18 |
| #20 | #3 and #9 and #19 | #3 and #9 and #19 |
| #21 | (#20) and ((PT=shourei houkoku・jirei nozoku) AND (PT=kaigiroku nozoku)) | (#20) and ((PT=excluding case reports) AND (PT=excluding conference proceedings)) |
| #22 | (#21) and (PT=kaisetsu, sousetsu, zusetsu, Q&A, kougi) | (#21) and (PT=review, overview, illustrated explanation, Q&A, lecture) |
| #23 | #21 not #22 | #21 not #22 |
| #24 | ekigaku teki houhou/TH | Epidemiologic methods/TH |
| #25 | ekigaku/TA | Epidemiology/TA |
| #26 | #24 or #25 | #24 or #25 |
| #27 | #20 and #26 | #20 and #26 |
| #28 | #23 and #26 | #23 and #26 |
| #29 | nihon/AL | Japan/AL |
| #30 | (nihon/TH or Japan/AL) | (Japan/TH or Japan/AL) |
| #31 | #29 or #30 | #29 or #30 |
| #32 | #28 and #31 | #28 and #31 |
